# Mosaic Deletions of Known Genes Explain Skeletal Dysplasias With High and Low Bone Mass

**DOI:** 10.1002/jbm4.10660

**Published:** 2022-07-05

**Authors:** Mari Muurinen, Fulya Taylan, Symeon Tournis, Jesper Eisfeldt, Alexia Balanika, Heleni Vastardis, Sirpa Ala‐Mello, Outi Mäkitie, Alice Costantini

**Affiliations:** ^1^ Research Program for Clinical and Molecular Metabolism University of Helsinki Helsinki Finland; ^2^ Children's Hospital University of Helsinki and Helsinki University Hospital Helsinki Finland; ^3^ Folkhälsan Research Center Helsinki Finland; ^4^ Department of Molecular Medicine and Surgery and Center for Molecular Medicine Karolinska Institutet Stockholm; ^5^ Department of Clinical Genetics Karolinska University Hospital Stockholm Sweden; ^6^ Laboratory for the Research of Musculoskeletal System "Th. Garofalidis," Medical School National and Kapodistrian University of Athens, KAT Hospital Athens Greece; ^7^ Department of Computed Tomography Asklepeion Voulas Hospital Athens Greece; ^8^ Department of Orthodontics, School of Dentistry National and Kapodistrian University of Athens Athens Greece; ^9^ Department of Clinical Genetics Helsinki University Hospital Helsinki Finland

**Keywords:** AMER1, MOSAICISM, RUNX2, SKELETAL DYSPLASIA, WHOLE‐GENOME SEQUENCING

## Abstract

Mosaicism, a state in which an individual has two or more genetically distinct populations of cells in the body, can be difficult to detect because of either mild or atypical clinical presentation and limitations in the commonly used detection methods. Knowledge of the role of mosaicism is limited in many skeletal disorders, including osteopathia striata with cranial sclerosis (OSCS) and cleidocranial dysplasia (CCD). We used whole‐genome sequencing (WGS) with coverage >40× to identify the genetic causes of disease in two clinically diagnosed patients. In a female patient with OSCS, we identified a mosaic 7‐nucleotide frameshift deletion in exon 2 of *AMER1*, NM_152424.4:c.855_861del:p.(His285Glnfs*7), affecting 8.3% of the WGS reads. In a male patient with CCD, approximately 34% of the WGS reads harbored a 3710‐basepair mosaic deletion, NC_000006.11:g.45514471_45518181del, starting in intron 8 of *RUNX2* and terminating in the 3′ untranslated region. Droplet digital polymerase chain reaction was used to validate these deletions and quantify the absolute level of mosaicism in each patient. Although constitutional variants in *AMER1* and *RUNX2* are a known cause of OSCS and CCD, respectively, the mosaic changes here reported have not been described previously. Our study indicates that mosaicism should be considered in unsolved cases of skeletal dysplasia and should be investigated with comprehensive and sensitive detection methods. © 2022 The Authors. *JBMR Plus* published by Wiley Periodicals LLC on behalf of American Society for Bone and Mineral Research.

## Introduction

Mosaicism refers to a state in which an individual has two or more populations of cells with different genotypes, caused by postzygotic *de novo* mutations.^(^ ^1^ ^)^ Mosaicism can occur in the germ cells, somatic cells, or both, depending on the timing and location of the mutation event. Monogenic disorders that occur in a mosaic state are often milder or have an atypical clinical presentation compared to the same disorder caused by a constitutional mutation and can be more challenging to diagnose. Although molecular diagnosis of mosaicism has become easier with advanced technologies that are able to detect even low‐level mosaicism, many mosaic disorders can still escape detection because of methodological limitations, such as insufficient sequencing depth.^(^ ^2^ ^)^

Sporadic cases of mosaicism have been described in skeletal dysplasia. Germline mosaicism has been reported in osteogenesis imperfecta type II, where multiple affected children have been born to unaffected parents.^(^ ^3^ ^)^ Stickler syndrome type II has been reported to result from mosaicism for a *COL11A1* mutation,^(^ ^4^ ^)^ and metatropic dysplasia from somatic mosaicism for a lethal *TRPV4* mutation.^(^ ^5^ ^)^ Somatic mosaicism for a common thanatophoric dysplasia mutation in *FGFR3* can cause atypical achondroplasia.^(^ ^6^ ^)^ In contrast to these disorders in which somatic mosaicism is rare, McCune‐Albright syndrome and the associated fibrous dysplasia is always caused by somatic activating mutations in *GNAS*.^(^ ^7^ ^)^ In many skeletal disorders, knowledge of the role of mosaicism is limited.

Osteopathia striata with cranial sclerosis (OSCS, OMIM: 300373) is an X‐linked dominant skeletal disorder caused by mutations in the *AMER1* gene, encoding the APC membrane recruitment protein 1, which plays a role in the Wnt/β‐catenin pathway. In heterozygous females, OSCS is characterized by longitudinal sclerotic striations of the long bones, cranial sclerosis, and craniofacial dysmorphism. In hemizygous males, the phenotype is typically more severe, varying from lethal to a milder survivable form.^(^ ^8^ ^)^ Somatic mosaicism of *AMER1* mutations has been reported in male patients with OSCS^(^ ^8^, ^9^, ^10^, ^11^ ^)^ and in one asymptomatic mother of OSCS patients.^(^ ^12^ ^)^ Germline mosaicism has also been reported.^(^ ^13^ ^)^

Cleidocranial dysplasia (CCD, OMIM: 119600) is an autosomal dominant skeletal disorder characterized by absent or hypoplastic clavicles, dental anomalies, and delayed closure of fontanelles. Small‐scale mutations in the gene encoding the Runt‐related transcription factor 2 (*RUNX2*), which plays a role in osteoblast and chondrocyte maturation, are identified in approximately 60% of CCD patients, whereas copy‐number variants (CNVs) in the same gene are responsible for approximately 10% of CCD cases.^(^ ^14^ ^)^ However, the rest of the CCD patients remain without a genetic diagnosis. Mosaicism of *RUNX2* mutations has been reported in asymptomatic mothers of CCD patients(^15^, ^16^ ^)^ and in a mildly affected father of a CCD patient.^(^ ^14^ ^)^

In this report, we present two cases of mosaicism in two patients affected by OSCS and CCD, expanding the pathomolecular mechanisms in these diseases and highlighting the novel methodological approaches.

## Subjects and Methods

The study was approved by the Ethics committee of the KAT General Hospital, Athens, Greece and the Research Ethics Committee of the Helsinki University Central Hospital, Finland. Written informed consents were obtained from all participants or their legal guardians. The study was conducted in accordance with the Declaration of Helsinki.

### Human subjects

Patient 1 was referred to the KAT hospital, Athens, Greece, for sclerosing bone dysplasia. In addition to the index patient, her healthy mother and healthy siblings were recruited to the study. The patient's medical history was reviewed, and a clinical examination was performed. Skeletal features were assessed by radiography, computed tomography (CT), and bone mineral density (BMD) measurement by dual‐energy X‐ray absorptiometry (DXA) (Lunar Prodigy; GE Lunar, Madison, WI, USA). Blood samples were obtained from all participating family members.

Patient 2, together with his healthy parents and two healthy siblings, was recruited from the Department of Clinical Genetics, Helsinki University Hospital, Finland. The patient's hospital records and radiographs were reviewed. Prior to recruitment, the genetic testing of the patient included sequencing of *ALPL* for suspected hypophosphatasia, sequencing and multiplex ligation‐dependent probe amplification (MLPA) analysis of *RUNX2* for suspected cleidocranial dysplasia, array–comparative genomic hybridization (CGH) analysis for copy number variants, and a gene panel testing for skeletal dysplasias (187 genes, including *RUNX2*). All genetic tests were negative for explaining the patient's phenotype. Peripheral blood samples were obtained from the patient and his family members.

### Whole‐genome sequencing and structural variant analysis

Paired‐end whole‐genome sequencing (WGS) (2 × 150‐basepair [bp] reads) was performed at the Science for Life Laboratory in Stockholm, Sweden. Genomic DNA (gDNA) was isolated from whole blood samples according to standard procedures and library preparation was carried out using the Illumina TruSeq polymerase chain reaction (PCR)‐free method (Illumina, San Diego, CA, USA). Sequencing was performed using the Illumina NovaSeq6000 System. The average sequencing coverage for the sample from patient 1 was 91×, whereas the average coverage for the samples from patient 2 and his four family members ranged from 42× to 63×. Single‐nucleotide variants (SNVs) were analyzed according to established in‐house pipelines.^(^ ^17^ ^)^ The sequencing data from patient 1 was filtered using a gene list, which only included 40 genes presently linked to high bone mass^(^ ^18^ ^)^ (Table S1). Variants with minor allele frequency <0.001 in GnomAD^(^ ^19^ ^)^ and SweGen,^(^ ^20^ ^)^ and with impact severity other than low in GEnome MINIng (GEMINI; https://gemini.readthedocs.io/en/latest/index.html) were considered in the analysis. The sequencing data from patient 2 and his family members was analyzed according to inheritance pattern and filtering the whole WGS data for variants with minor allele frequency <0.001 in GnomAD and SweGen, and with impact severity other than low in GEMINI. The variants are visualized using Integrative Genomics Viewer (IGV).

Structural variant (SV) analysis was performed using the FindSV pipeline. FindSV carries out variant detection using CNVnator V0.3.2^(^ ^21^ ^)^ and TIDDIT V2.0.0.^(^ ^22^ ^)^ Structural Variant Database (SVDB) (https://github.com/J35P312/SVDB) is used to merge the data from these variant callers and variant annotation is performed using Variant Effect Predictor (VEP) 92.^(^ ^23^ ^)^ Finally, the detected variants are filtered using a minor allele frequency <0.001 in SweGen as well as using the gene list from the latest Nosology of genetic skeletal diseases.^(^ ^18^ ^)^

### Sanger sequencing

Sanger sequencing was performed according to standard procedures using the BigDye™ Terminator v3.1 Cycle Sequencing Kit (Applied Biosystems [ABI], Foster City, CA, USA; #4337456). Samples were sequenced on a 3730 ABI sequencer and the electropherograms were visualized using 4Peaks version 1.7.2. Primer sequences are available upon request.

### Droplet digital PCR

Droplet digital PCR (ddPCR) was performed on gDNA extracted from whole blood samples. For each family, ddPCR was performed for the index patient, family members, and two commercially available human female and male gDNA control samples (Promega, San Luis Obispo, CA, USA; #G1521 and G1471). A no template control (NTC), in which purified water was added instead of gDNA, was included for each assay and all reactions were performed in duplicates.

Briefly, 33 ng of gDNA isolated from blood were used as input material for performing PCR using the ddPCR™ Supermix for Probes (No 2′‐Deoxyuridine, 5′‐Triphosphate [dUTP]) kit (BioRad, Hercules, CA, USA; #1863023). Each reaction contained a primer pair and two double‐quenched probes, one labeled with the HEX dye (5′ HEX/ZEN/3′IBFQ) to detect the wild‐type allele and the other labeled with the FAM dye (5′FAM/ZEN/3′IBFQ) to detect the mutant allele. The primers and probes used in this study are listed in Table S2 and the PCR conditions are described in Tables S3 and S4. Droplets were generated on the Automated Droplet Generator (BioRad; #1864101), PCR was performed using the Touch Real‐Time PCR Detection System (BioRad), and subsequently the target DNA was read QX200 on the Droplet Reader (BioRad; #1864003).

Each sample was run in triplicate. The QuantaSoft Analysis Pro v.1.0 (BioRad) was used to analyze the data. The threshold for true signal positivity was adjusted based on the signal in the control samples. Samples with <10,000 droplets were excluded from the analysis. The level of mosaicism is expressed as a ratio of the number of deletion‐positive droplets to the total number of DNA‐containing droplets (mutant + wildtype).

### Statistical analysis

A Poisson distribution with 95% confidence interval (CI) was used to determine the number of positive and negative droplets.

## Results

### Clinical findings

#### Patient 1

**Fig. 1:**
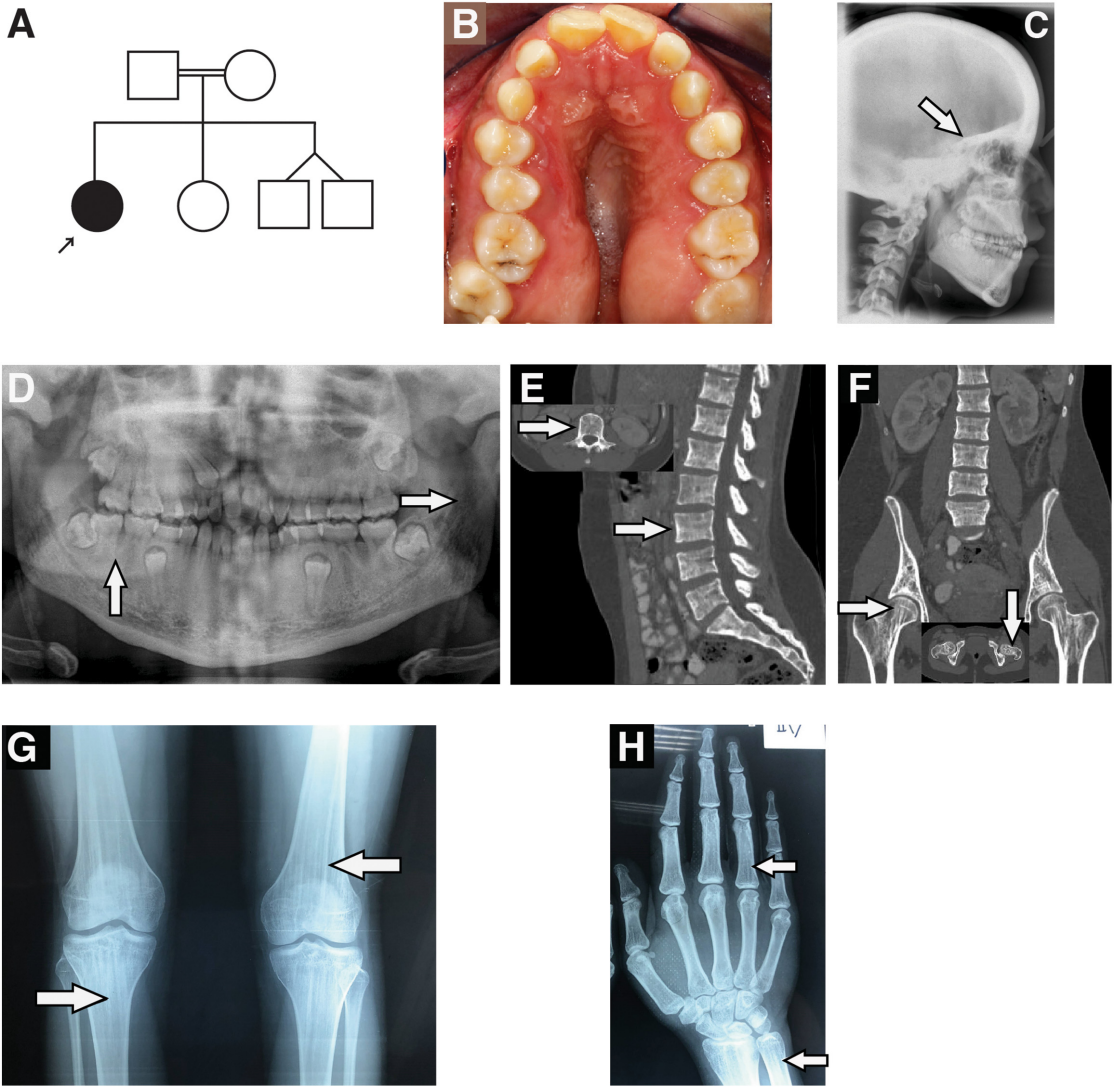
(*A*) Pedigree of the family of patient 1. (*B*) Occlusal view of the maxilla revealing anterior open bite and cross bite, the Ω‐shape, significant stenosis, thick alveolar ridges, and over‐retention of the upper right primary canine. (*C*) Lateral cephalometric radiograph at the age of 18 years showing thickening of the cranial vault and cranial base (arrow). (*D*) Panoramic radiograph at the age of 18 years demonstrating linear densities of the ramus of the mandible and periapical bone sclerosis (arrows). (*E*, *F*) Abdominal CT (sagittal and coronal view) at the age of 19 years showing trabecular thickening of the lumbar vertebrae (arrows, panel E) and linear striation of the femoral necks (arrows, panel F). (*G*,*H*) Frontal knee and hand radiographs at the age of 19 years showing regular linear sclerosing bands extending vertically in the metaphysis of both distal femora and proximal tibia (arrows, panel G), both distal ulna and radius and in the phalangeal diaphysis (arrows, panel H).

A 19‐year‐old female (weight 56 kg, height 157 cm), evaluated for a “sclerosing bone dysplasia” was born to healthy consanguineous parents (second cousins) at the 38th week of gestation (birth weight 2750 g); her siblings were healthy (Fig. 1*A*). She was diagnosed with a hearing impairment and delayed eruption of primary teeth at 4 years and underwent multiple extractions of primary teeth at 7 years, allowing eruption of some permanent teeth. At 12 years, and again at 14 years, she underwent tonsillectomy and bilateral tympanostomy due to chronic otitis media. However, progressive conductive and sensorineural hearing loss necessitated hearing aids. She was also diagnosed with mild intellectual disability mainly due to hearing loss.

Clinical examination at 19 years showed wide nasal bridge, frontal bossing, ocular hypertelorism, long lower face, angular palate, and dental anomalies (Fig. 1*B*). She had macrocephaly (head circumference 61.5 cm, >+2.0 standard deviation [SD]) and diffuse sclerosis, especially of the cranial base, was noted (Fig. 1*C*). Panoramic radiography showed sclerosing densities (radiopacities) in the ramus of the mandible and in the periapical region of the teeth and retention of certain primary teeth (Fig. 1*D*). The permanent dentition showed anterior open bite, posterior cross bite, and maxillary stenosis. In addition, other findings included vertical trabecular thickening of the lumbar vertebral bodies (Fig. 1*E*) and dense linear striations at femoral necks (Fig. 1*F*). Skeletal radiographs revealed dense linear striations in the metaphysis extending into the epiphyseal area of the tubular bone (Fig. 1*G*,*H*). BMD *Z*‐score was +3.0 for the lumbar spine, +1.6 for whole body less head, and +4.7 for whole body (WB‐BMD).

#### Patient 2

**Fig. 2:**
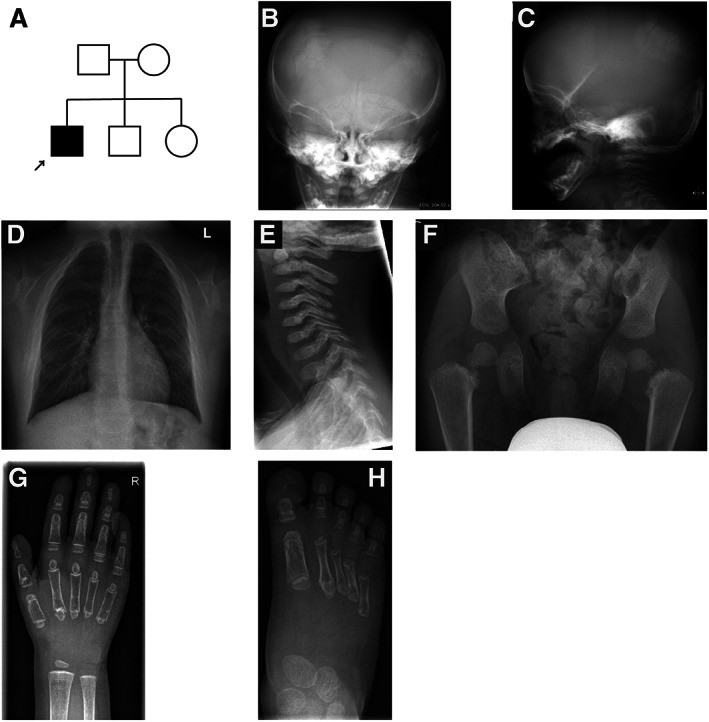
(*A*) Pedigree of the family of patient 2. (*B*,*C*) Radiographs of the skull at 1 year of age showing poor ossification of the skull and Wormian bones in the parietal and occipital regions. (*D*) Radiograph of the thorax at age of 12 years showing missing clavicles. (*E*) Radiograph of the neck at the age of 4 years showing platyspondyly of the cervical vertebrae, missing anterior arch of C1, and round, small dens. (*F*) Pelvic radiograph at the age of 4 years showing delayed bone development, narrow ilia, short femoral necks, and unossified pubic rami. (*G*) Radiograph of the hand at the age of 3 years showing unusual shape of the middle phalanges, osteopenia, and delayed ossification of the carpal bones. (*H*) Radiograph of the foot at the age of 3 years showing unusual shape of the proximal phalanges.

A currently 14‐year‐old male was born to healthy nonconsanguineous parents at the 41st week of gestation (birth weight 4000 g, birth length 49 cm [−1.1 SD], head circumference 34.5 cm [−0.6 SD]) (Fig. 2*A*). His siblings were healthy. Short limbs were observed on prenatal ultrasound at 20th week of gestation. Prenatal testing identified a normal male karyotype. At birth, dysmorphic facial features and severely abnormal development of the skull bones were evident on clinical examination and radiographs (Fig. *2B‐C*). The patient had hypoplastic vertebrae, short ribs, and only partially mineralized clavicles and pubic rami (Fig. *2D‐F*). Serum alkaline phosphatase was low (49–57 U/L; reference 60–275 U/L) during the neonatal period, and repeatedly below normal range during childhood. Initially, either hypophosphatasia or cleidocranial dysplasia was suspected, but later cleidocranial dysplasia was considered the most likely diagnosis. His height *Z*‐score progressively deteriorated from −2.2 at 4 months to −4.9 at 24 months. He had obstructive sleep apnea and used continuous positive airway pressure therapy (CPAP) during infancy. He had a very low nasal bridge, small nose with anteverted nares, midface hypoplasia, and hypertelorism. His supraorbital region was low, causing exophthalmos. He had increased lumbar lordosis and abnormally shaped thoracal vertebrae (Fig. *2D‐E*). BMD was low (lumbar spine *Z*‐score −5.4 at 6 years, left hip neck *Z*‐score −5.2 at 6 years, and lumbar spine *Z*‐score −3.1 at 12 years), but he sustained no fractures. Femoral metaphyses were abnormal. Coxa vara was surgically corrected. A delayed ossification of the carpal bones and an abnormal shape of the phalanges were noticed (Fig. *2G‐H*). He also had misalignment of ankles and elbows. Shoulders were narrow, he only had 11 pairs of ribs, and his scapulae were abnormal in shape. At school age, he was diagnosed with hearing loss necessitating the use of hearing aids. He had multiple supernumerary teeth and hypoplastic enamel. At 11 years of age, he had short stature (−4.5 SD), his primary teeth were still intact, and fontanelles were widely open.

### Molecular findings

**Fig. 3:**
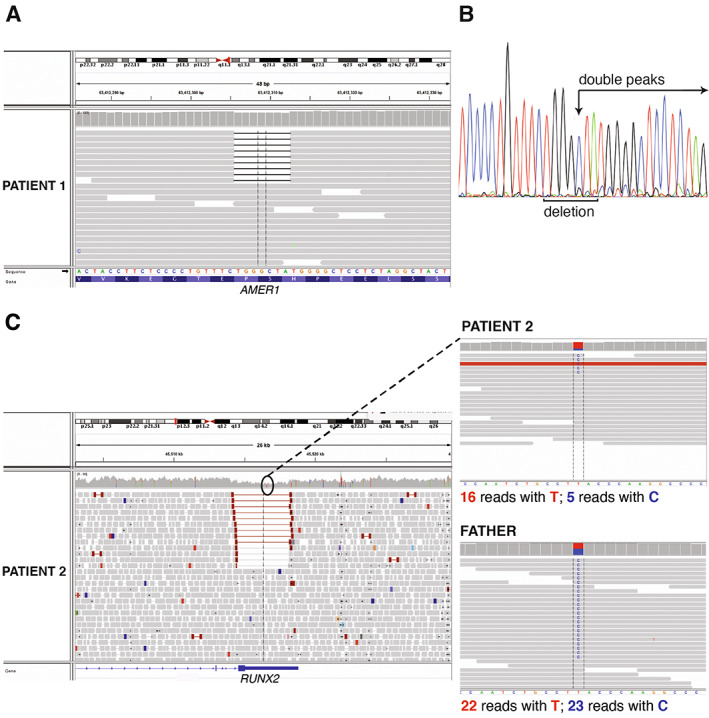
(*A*) Snapshot of the WGS reads visualized using the Integrative Genomics Viewer tool showing the *AMER1* region affected by the mosaic 7‐nucleotide deletion in patient 1. (*B*) Sanger electropherogram of the region spanning the mosaic deletion showing a reading frameshift (small double peaks indicated by the arrow). (*C*) Snapshot of the WGS reads visualized using the Integrative Genomics Viewer tool showing the *RUNX2* region affected by the mosaic 3710‐bp deletion in patient 2. On the right, a single nucleotide polymorphism within the region affected by the mosaic deletion. Patient 2 has inherited this change from his healthy father. A skewed ratio of reads with the wild‐type allele (T) and alternative allele (C) at this locus confirms the presence of a mosaic deletion in the index patient.

Because patient 1 had a clinical diagnosis of OSCS, the genetic screening was first done by Sanger sequencing for *AMER1*, which failed to identify any causative variant. WGS was then performed in the index patient (Fig. 1*A*), targeting the analysis to genes that have been linked to high bone mass, and identified a mosaic 7‐nucleotide (nt) frameshift deletion in the last exon (exon 2) of *AMER1*: NM_152424.4: c.855_861del:p.(His285Glnfs*7) (Fig. 3*A*). The deletion was present in 8.3% of the WGS reads (nine out of the total number of 108 reads). Reevaluation of the Sanger sequencing data showed evidence of low‐level mosaicism (Fig. 3*B*).

For patient 2, because previously done Sanger sequencing, MLPA, and next‐generation sequencing (NGS) gene panel analysis had excluded *RUNX2* variants, WGS in the index patient and his family members (Fig. 2*A*) was performed. In the WGS analysis, no single‐nucleotide variant or small insertion/deletion that could explain the patient's phenotype was detected. In the SV analysis of WGS data, a 3710‐bp mosaic deletion in *RUNX2* was identified: NC_000006.11:g.45514471_45518181del (Fig. 3*C*). The breakpoints of this deletion fell into intron 8 and the 3′ untranslated region (UTR) of *RUNX2*. Approximately 34% of the reads supported the deletion, indicating that the deletion was present in mosaic state.

**Fig. 4:**
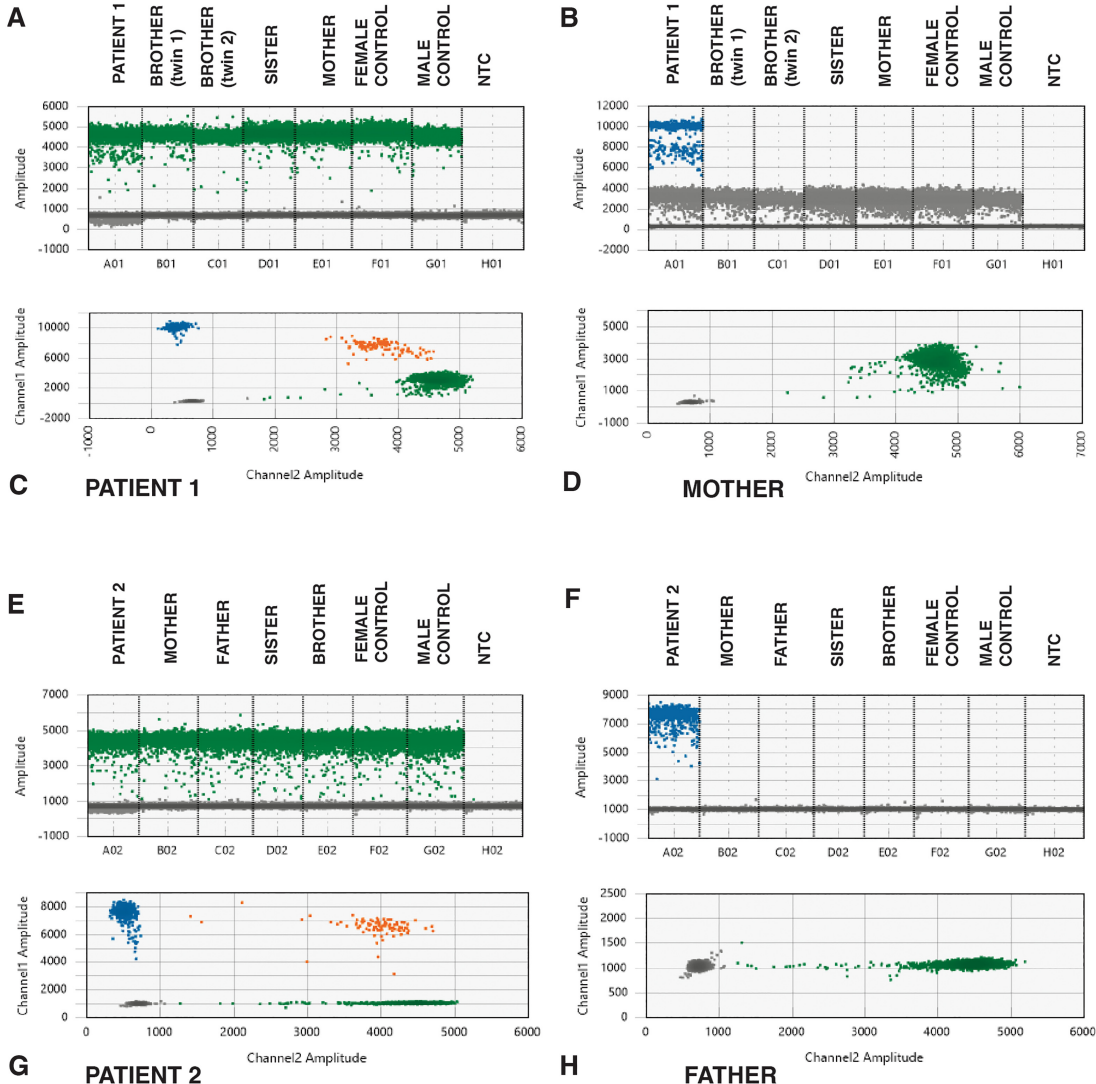
ddPCR results for patient 1 (*A*–*D*) and patient 2 (*E*–*H*). Droplets detecting the allele with the deletion are marked in blue, droplets detecting the wild‐type allele are marked in green, and droplets with both the deletion and the wild‐type sequence are shown in orange. The negative droplets are displayed in gray. (*A*,*B*) 1D plots showing the droplets with the wild‐type and deleted sequence in exon 2 of *AMER1* in the patient, her unaffected family members, two controls, and the NTC sample. Droplets with the deletion were only found in the index patient. (*C*,*D*) 2D plots of the droplets in patient 1 and her mother. Only the wild‐type sequence is detected in the mother's sample. (*E*,*F*) 1D plots showing the droplets with a wild‐type sequence in exon 4 and the droplets with the sequence spanning the breakpoints of the deletion in patient 2, his unaffected family members, two controls, and the NTC sample. Signal from the probe spanning the breakpoints of the deletion in *RUNX2* was only detected in the index patient. (*G*,*H*) 2D plots of the droplets in patient 1 and his father. Only droplets with probes detecting the wild‐type sequence were found in the father's sample. NTC = no‐template control.

The level of mosaicism in both patients’ blood samples was further ascertained by ddPCR because this is a highly sensitive method compared to WGS. ddPCR determined the percentage of droplets harboring the deletion as 11% and 32% in patient 1 and patient 2, respectively. As expected, none of their family members showed any mosaicism for the investigated variants (Fig. 4*A–H*).

## Discussion

Using WGS and ddPCR, we have identified and validated two cases of mosaic mutations leading to two types of skeletal dysplasias with abnormal bone mass, OSCS and CCD. Both patients had phenotypes that directed genetic testing towards the causative genes typically identified in their disorders, *AMER1* in OSCS and *RUNX2* in CCD. Initial efforts in identifying the mutations in *AMER1* and *RUNX2* were unsuccessful because of inefficiencies of the employed methods in identifying mosaicism and structural variants. Both array‐CGH and MLPA missed the mosaic deletion in patient 2. Although these methods are widely used to detect CNVs, they are limited by probe density, CNV size, and experimental design. Moreover, the ability to detect low‐grade mosaicism is limited for MLPA,^(^ ^24^ ^)^ whereas array‐CGH usually detects mosaic CNVs when the variant is present in >10% of the total cell population.^(^ ^1^ ^)^ By using a more comprehensive technology, WGS, we were able to identify mosaicism in both patients. Final validation of the results was done with ddPCR, a technology that can detect mosaicism with a sensitivity as low as 0.001%.^(^ ^25^ ^)^ To our knowledge, the *AMER1* deletion in patient 1 is the first mosaic mutation described in a female patient with a diagnosis of OSCS, and the mosaic *RUNX2* deletion in patient 2 is the second description of a mosaic deletion in an individual affected by CCD.

The percentage of DNA molecules with the *AMER1* deletion in the blood of patient 1 is estimated at around 11%, thus meaning that the level of mosaicism, which is the percentage of cells harboring the mutation, is approximately 22%. The level of mosaicism can differ between different tissues and modify the observed phenotype and disease severity. The phenotype of the patient, with linear striations of long bones, cranial sclerosis, hypertelorism, frontal bossing, wide nasal bridge, dental anomalies, hearing loss, and mild intellectual disability, is comparable to the phenotype of patients with constitutional *AMER1* mutations and OSCS. It is therefore likely that the mutation is not only present in the bone tissue of the patient but also the level of mosaicism in bone can be equal to or higher than the detected level in the blood, causing the observed phenotype. Because *AMER1* is on the X chromosome, skewed X‐inactivation in a female patient may also play a part in affecting the clinical phenotype. However, we had no access to samples that would have enabled us to study skewed X‐inactivation and evaluate its potential effect on the phenotype of our patient. The previously reported female case of mosaic deletion of Xq11.1 involving the entire *AMER1* gene (also known as *WTX* and previously as *FAM123B*) had a detectable mosaicism in blood (17%) and buccal (15%) samples. She was the mother of OSCS patients but was not reported to have OSCS herself.^(^ ^12^ ^)^ The presence of a high percentage of cells harboring the *AMER1* deletion in the bone tissue of the patient could potentially explain why she is affected by OSCS despite having a relatively low level of mosaicism in the blood comparable to the unaffected subject reported by Wilson and colleagues.^(^ ^12^ ^)^

Patient 2 had characteristic features of CCD, but the delay of bone mineralization was much more severe than typically seen in CCD, and the phenotype initially resembled hypophos‐phatasia. The patient also had decreased levels of alkaline phosphatase and markedly reduced BMD. Similar patients with severe CCD and features of hypophosphatasia have been described.^(^ ^26^, ^27^ ^)^ The patient with severe CCD and hypophos‐phatasia described by El‐Gharbawy and colleagues^(^ ^27^ ^)^ had a 50–70‐kb heterozygous deletion in the C‐terminal region of *RUNX2*, outside of the RUNT domain. The deletion partially overlaps with the mosaic deletion of our patient. Although mosaicism can often cause a milder phenotype than a constitutional mutation, the severity of the patient's symptoms is probably explained by the deletion in a location that has been reported in other similar severe cases of CCD. Mosaic mutations of *RUNX2* have not been previously described in CCD patients, but because ~32% of DNA molecules in our patient's blood harbor the deletion, our finding is consistent with the report of a mouse model where ≥30% reduction in Runx2 wild‐type messenger RNA (mRNA) level produces a CCD phenotype.^(^ ^28^ ^)^ Our results suggest that the level of *RUNX2* mosaicism in patient 2's blood cells is 64%.

In our study, the identification of mosaicism levels was limited to DNA extracted from the blood samples of the patients and their family members. We are therefore unable to determine the extent of mutations in other tissues. Considerable variation may exist between different tissue types and the level of mosaicism detected in blood does not necessarily reflect the level of mosaicism in other tissues. Optimally, in skeletal dysplasia the level of mosaicism should be studied in bone tissue, but such samples are seldomly available. Buccal swabs are easier to obtain, but the cellular content of a buccal swab includes a variable mixture of lymphocytes and epithelial cells,^(^ ^29^ ^)^ complicating the interpretation of the level of mosaicism in such samples. Skin biopsies would have been more suitable for the study. However, contacting the families for a second sample collection was unfortunately not possible. Depending on the timing of the mutagenesis during embryonic development, in addition to the identified somatic mosaicism it is also possible that the patients could have germline mosaicism, indicating a risk for their future pregnancies. Furthermore, in some known skeletal dysplasias caused by somatic mutations, such as melorheostosis (OMIM: 155950) and fibrous dysplasia (OMIM: 174800), mosaicism is only detected in bone tissue, whereas the mutation is absent in blood.

In conclusion, we identified two novel mosaic mutations in *AMER1* and *RUNX2*, leading to OSCS and CCD phenotypes, respectively. Our study indicates that mosaicism of mutations in known genes of skeletal disorders can lead to phenotypes that resemble disorders caused by constitutional mutations of the same genes. Therefore, as the novel molecular technologies provide us more sensitive detection levels of mutations, it is likely that more cases like these will be described in the future. In CCD, with around 30% of patients lacking a genetic diagnosis, mosaicism detection can potentially increase the diagnostic yield. Considering mosaicism and structural variants, unsolved cases with a clear clinical diagnosis, as suggested by our study, can benefit from reanalysis with more comprehensive and sensitive methods, such as WGS with high sequencing coverage in combination with ddPCR. Exome sequencing, a popular method often used instead of WGS, may also not be sufficient to detect mosaic CNVs due to dlimitations such as uneven coverage of sequencing reads over the exons. In patients with a milder presentation of symptoms due to mosaicism, diagnosing a patient is even more challenging. In these cases, using appropriate methods to detect mosaicism becomes crucial in finding the correct genetic diagnosis.

## Author contributions

**Mari Muurinen:** Conceptualization; data curation; formal analysis; investigation; resources; writing – original draft; writing – review and editing. **Fulya Taylan:** Conceptualization; data curation; formal analysis; investigation; methodology; resources; validation; writing – original draft; writing – review and editing. **Symeon Tournis:** Data curation; investigation; resources; writing – review and editing. **Jesper Eisfeldt:** Formal analysis; methodology; writing – review and editing. **Alexia Balanika:** Data curation; writing – review and editing. **Heleni Vastardis:** Data curation; writing – review and editing. **Sirpa Ala‐Mello:** Data curation; investigation; writing – review and editing. **Outi Makitie:** Conceptualization; data curation; formal analysis; funding acquisition; investigation; project administration; resources; supervision; validation; writing – original draft; writing – review and editing. **Alice Costantini:** Conceptualization; data curation; formal analysis; funding acquisition; methodology; project administration; resources; software; supervision; validation; writing – original draft; writing – review and editing.

## Conflicts of Interest

The authors have no conflicts of interest to declare.

## Supporting information


**Table S1.** List of genes linked to high bone mass and screened in patient 1.
**Table S2**. Primers and probes used for ddPCR.
**Table S3**. ddPCR mastermix.
**Table S4**. PCR conditions for ddPCR.

## Data Availability

The data supporting the results are included in the main text and in the Supplemental Material. Additional data are available from the corresponding author upon request.
