# Supplementary material for: Mosaic Deletions of Known Genes Explain Skeletal Dysplasias With High and Low Bone Mass
Source: JBMR Plus. 2022 Jul 5;6(8):e10660. doi: 10.1002/jbm4.10660 (PMC9382864; doi:10.1002/jbm4.10660)
Supplement: Supplementary file 1 — Table S1. List of genes linked to high bone mass and screened in patient 1. Table S2. Primers and probes used for ddPCR. Table S3. ddPCR mastermix. Table S4. PCR conditions for ddPCR. [file JBM4-6-e10660-s001.docx]

**Supplemental Information**

Supplemental information about the list of genes analyzed in patient 1 and the reagents and conditions used for performing Droplet Digital PCR (ddPCR) are summarized in Supplemental Tables 1-4.

**Supplemental Table 1. List of gene linked to high bone mass and screened in patient 1.**

| **Gene names** | | | |
| --- | --- | --- | --- |
| *AMER1* | *FERMT3* | *LRP5* | *SLC29A3* |
| *ANKH* | *FGF23* | *LRRK1* | *SLCO2A1* |
| *CA2* | *GALNT3* | *MAP2K1* | *SNX10* |
| *CLCN7* | *GJA1* | *MTAP* | *SOST* |
| *COL1A1* | *GJA1* | *OSTM1* | *TBXAS1* |
| *CSF1R* | *HPGD* | *PLEKHM1* | *TCIRG1* |
| *CTSK* | *IKBKG* | *POLR3B* | *TGFB1* |
| *DHCR24* | *KL* | *PTDSS1* | *TNFRSF11A* |
| *DLX3* | *LEMD3* | *PTHR1* | *TNFRSF11B* |
| *FAM20C* | *LRP4* | *SFRP4* | *TNFSF11* |

**Supplemental Table 2. Primers and probes used for ddPCR.**

| **Name of the probe/primer** | **Targeted gene, region** | **Sequence** | **Strand** | **Length** | **Tm** | **GC %** |
| --- | --- | --- | --- | --- | --- | --- |
| AMER1_wt_probe | *AMER1*, exon 2/2 | CCCCTGTTTCTGGGCTATGGGGCTCC | sense | 26 | 64.1 | 65.4 |
| AMER1_mut_probe | *AMER1*, exon 2/2 | CCCCTGTTTCTGGGGCTCCTCTAGGCT | reverse | 27 | 64.4 | 63 |
| AMER1_wt/mut_F | *AMER1*, exon 2/2 | CATTGGGTGGGTTTACCTC | sense | 20 | 60.4 | 55 |
| AMER1_wt_R | *AMER1*, exon 2/2 | CATGTGCAACCCAAGCCT | reverse | 18 | 60.5 | 55.6 |
| AMER1_mut_R | *AMER1*, exon 2/2 | CATGTGCAACCCAAGCCT | reverse | 18 | 60.5 | 55.6 |
| RUNX2_wt_probe | *RUNX2*, exon 4/9 | CTGAGCTCCGGAATGCCTCTGCTGTT | sense | 26 | 62.6 | 57.7 |
| RUNX2_mut_probe | *RUNX2*, spans two breakpoints of the deletion | CCTTTATGGGAAAGCTTTAAGGATTCCCTC | sense | 30 | 57.6 | 43.3 |
| RUNX2_wt_F | *RUNX2*, exon 4/9 | ATGGGACTGTGGTTACTG | sense | 18 | 55.6 | 50 |
| RUNX2_wt_R | *RUNX2*, exon 4/9 | CACAAATCTCAGATCGTTGA | reverse | 20 | 55.6 | 40 |
| RUNX2_mut_F | *RUNX2*, intron 8-9 | CATTTGAAGGTCTGTCTG | sense | 18 | 55.2 | 44.4 |
| RUNX2_mut_R | *RUNX2*, exon 9/9 | TGTTTCCATGTATTAACCTG | reverse | 20 | 55.5 | 35 |

wt= wildtype; mut=mutation; F= forward; R= reverse. Probes are marked with green and primers are marked with black.

**Supplemental Table 3. ddPCR mastermix.**

|  | **Concentration** | **Volume** |
| --- | --- | --- |
| **ddPCR Supermix for Probes (No dUTP)** | 2X | 10 μl |
| **primer F** | 4.5 μM (final 900 nM) | 4 μl |
| **primer R** | 4.5 μM (final 900 nM) | 4 μl |
| **probe mut** | 6.2 μM (final 260 nM) | 0.8 μl |
| **probe wt** | 6.2 μM (final 260 nM) | 0.8 μl |
| **DNA** | 30 ng/μl | 1 μl |
| **Total Volume** |  | 20.6 μl |

**Supplemental Table 4. PCR conditions for ddPCR.**

|  | ***AMER1*, *RUNX2*** | ***AMER1*, *RUNX2*** | ***AMER1*** | ***RUNX2*** | ***AMER1*, *RUNX2*** | ***AMER1*, *RUNX2*** |
| --- | --- | --- | --- | --- | --- | --- |
| **# of cycles** | 1 | 40 | 40 | 40 | 1 | 1 |
| **Temperature (°C)** | 95 | 94 | 60 | 52,5 | 98 | 4 |
| **Time** | 10 min | 30 sec | 1 min | 1 min | 10 min | 15 min |
|  | Enzyme activation | Denaturation | Annealing/Extension | | Enzyme deactivation | Hold |
